# Finite Element Modeling of Tensile Deformation Behaviors of Iron Syntactic Foam with Hollow Glass Microspheres

**DOI:** 10.3390/ma10101201

**Published:** 2017-10-19

**Authors:** Yi Je Cho, Wookjin Lee, Yong Ho Park

**Affiliations:** 1 Department of Materials Science and Engineering, Pusan National University, Busandaehak-ro 63beon-gil 2, Busan 46241, Korea; yhpark@pusan.ac.kr; 2 Korea Institute of Industrial Technology (KITECH), Namyangsan 1-gil 14, Yangsan 50635, Korea

**Keywords:** iron syntactic foam, glass microsphere, representative volume element, finite element method, tensile deformation

## Abstract

The elastoplastic deformation behaviors of hollow glass microspheres/iron syntactic foam under tension were modeled using a representative volume element (RVE) approach. The three-dimensional microstructures of the iron syntactic foam with 5 wt % glass microspheres were reconstructed using the random sequential adsorption algorithm. The constitutive behavior of the elastoplasticity in the iron matrix and the elastic-brittle failure for the glass microsphere were simulated in the models. An appropriate RVE size was statistically determined by evaluating elastic modulus, Poisson’s ratio, and yield strength in terms of model sizes and boundary conditions. The model was validated by the agreement with experimental findings. The tensile deformation mechanism of the syntactic foam considering the fracture of the microspheres was then investigated. In addition, the feasibility of introducing the interfacial deboning behavior to the proposed model was briefly investigated to improve the accuracy in depicting fracture behaviors of the syntactic foam. It is thought that the modeling techniques and the model itself have major potential for applications not only in the study of hollow glass microspheres/iron syntactic foams, but also for the design of composites with a high modulus matrix and high strength reinforcement.

## 1. Introduction

Metal matrix syntactic foams are a new class of materials that combine the properties of particle-reinforced metal matrix composites and metal foams. These composites were developed to overcome the limitations of the mechanical properties of closed-cell metal foams for load-bearing applications [1]. While they have a higher density, metal matrix syntactic foams possess mechanical properties superior to those of the conventional foams. Many metals have been adapted for matrix materials, and various hollow microsphere materials selected for their size could provide different properties [2,3,4,5].

As a first step for developing new metal syntactic foams, many studies have been conducted to estimate mechanical properties based on microstructure property relationships [6,7,8,9,10,11]. Classical homogenization approaches for analytical modeling [6,7,8,9,10], based on the Eshelby solution for a spatially uniform strain over an ellipsoidal homogeneous inclusion, were unable to accurately predict the elastic properties. This is because void phases inside the hollow microspheres were contained inside a specific phase, while the matrix was a continuous phase. These models only provided ranges of mechanical property values at a specific volume fraction of the microspheres [11]. Thereafter, the heterogeneity of the microstructure was considered to develop more accurate analytical models [11,12,13,14]. In addition, a model for estimating the compressive strength of the syntactic foams was proposed, considering the load partitioning effect of the hollow microspheres [15]. However, the absence of the microstructure information limits the capability of these models to provide accurate predictions. For instance, the clustering, fractured shells, and interfacial debonding of the hollow microspheres can degrade mechanical properties [16,17,18]. Furthermore, tensile properties cannot be estimated by these analytical models.

Numerical techniques, especially the finite element method (FEM), have received attention for achieving a higher resolution of stress and strain fields than is feasible with analytical models. The estimation of syntactic foam properties can be powerfully enhanced by the application of numerical models suited to overcoming the limitations of the analytical models, notably in relation to microstructure details. Two typical models are the unit cell and representative volume element (RVE). The unit cell model can compute mechanical properties in a short time. However, it is unfeasible to incorporate the spatial distribution and interactions of the hollow microspheres due to the periodicity of the model. In comparison, the RVE model can overcome such limitations of the unit cell model, and it has been successfully used for modeling of the syntactic foams because it assumes that all constituents are contained with significant enough quantities in the model to accurately represent the material behaviors [1,11,16,19,20,21,22]. However, RVE models had only been used thus far to describe the behaviors of polymer matrix syntactic foams [11,16,19,20,21,22]. With respect to polymer syntactic foams, the elastic modulus and fracture strength of the ceramics and glasses composing the microspheres are mostly greater than those with respect to polymers. The case of the metal syntactic foams with those microspheres is the opposite of that of the polymer, owing to a higher elastic modulus and elongation of the metal. These differences between polymer and metal syntactic foams can cause differences in mechanical properties and deformation mechanisms. Although a three-dimensional RVE model was previously developed for the metal syntactic foams, only the elastic modulus was predicted [1]. To the best of our knowledge, none of the reported RVE models consider the plastic deformation of the metal syntactic foams as well as the fracture of the microspheres. An in-depth analysis of these behaviors is vital since they play a key role in the deformation of the metal syntactic foams.

The aim of the present study was to develop an RVE model to represent the elastoplastic tensile behaviors of iron syntactic foams with glass microspheres. Models of different sizes were generated for numerical analysis, and validated by a statistical method and by a comparison between numerical, theoretical, and experimental results. Using the models, the deformation mechanism with the fracture behaviors of the glass microspheres was investigated.

## 2. Material for Modeling

A metal matrix syntactic foam consisting of an iron matrix and hollow glass microspheres was chosen as a material for the numerical analysis. Gas-atomized pure iron powder (d_average_ = 3 μm, FEE12PB, Kojundo Chemical Laboratory Co., LTD (Sakado, Saitama, Japan) [23]) and hollow soda-lime borosilicate glass microspheres (d_average_ = 18 μm, with shell thickness of 0.806 μm, iM30K, 3M™ [24]) were homogeneously mixed with a weight ratio of 95:5 by a 3D mixer for 48 h. Green bodies were fabricated by 150 MPa uniaxial pressing and sintered at 900 °C for 1 h in flowing H_2_ gas with a heating rate of 5 °C/min.

Microstructures of the samples were observed using optical microscope and scanning electron microscope. Density was measured by Archimedes′ method. Uniaxial tensile tests were conducted six times to evaluate mechanical properties using a universal testing machine with a constant strain rate of 10^−3^ s^−1^. The sintered samples were cut into small-sized tensile specimens with a plate shape according to the KS B0801 standard [25]. The gauge length, width, radius of fillet, and length of the reduced section were 9.8, 2, 4, and 13.2 mm, respectively. Strain gauges were attached to the specimens for elongation measurements.

**Figure 1:**
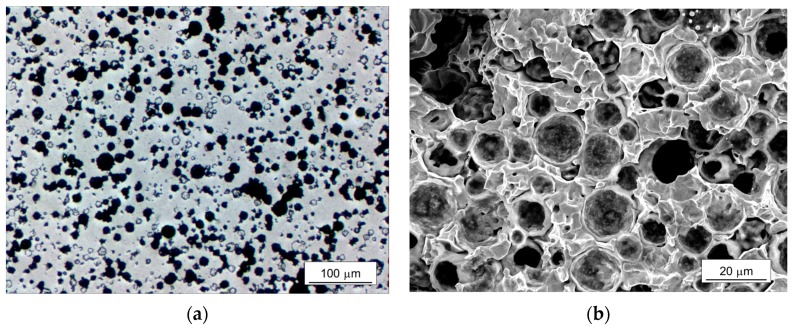
(**a**) Optical microscope image of the microstructure; (**b**) scanning electron microscope image of a freeze-fractured cross section of the iron syntactic foam with 5 wt % hollow glass microspheres.

Figure 1 shows a microstructure of the sintered iron syntactic foam with 5 wt % microspheres. In Figure 1a, the microspheres retained spherical shapes and were homogeneously distributed in the matrix. Some of them were filled with matrix, which occurred during the polishing of the surface. A freeze-fractured cross section in Figure 1b shows more clearly the surviving microspheres. Partially broken shells filled by the iron powders were observed, which would have been caused during the compression of the mixture of the iron powder and microsphere. Although softening of the glass occurs around 600 °C and the sintering was conducted at 900 °C, the shape of the microspheres remained spherical, indicating that there was no pronounced deformation or degradation of the microspheres during the sintering process. It also indicates that a proper compacting pressure was applied for the fabrication of the green body without significant damage to the microspheres. The interfaces between the iron matrix and microspheres were in well-bonded states. The density and relative density compared to theoretical density of the iron (7.87 g·cm^−3^) were 5.38, and 0.68 g·cm^−3^, respectively. The measured density was 9% higher compared to the theoretical density of the syntactic foam calculated by a simple rule of mixture (=~4.90 g·cm^−3^), which would have been induced by the broken microspheres. The models for simulations were reconstructed based on this microstructure.

## 3. Finite Element Modeling

### 3.1. Modeling Procedures

**Figure 2:**
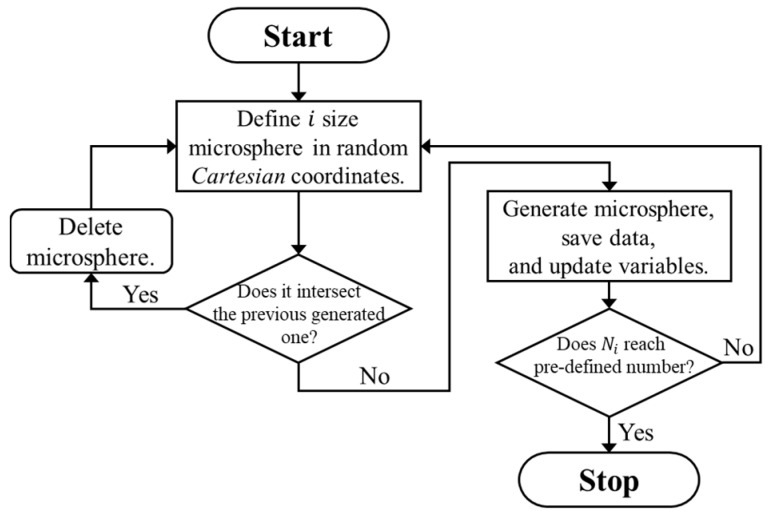
Schematic flow chart of the random sequential adsorption algorithm, where i and $N_{i}$ are the diameter and the number of glass microspheres, respectively.

**Figure 3:**
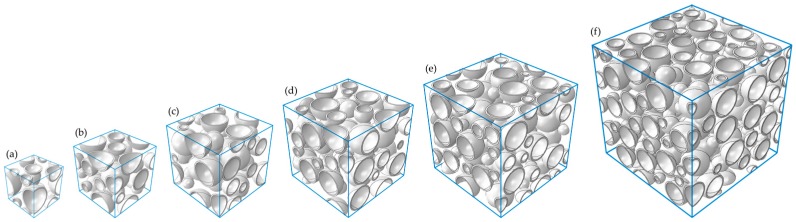
Typical generated models with edge lengths of (**a**) 25 μm; (**b**) 35 μm; (**c**) 45 μm; (**d**) 55 μm; (**e**) 65 μm; and (**f**) 85 μm.

On the basis of the observed microstructures of the iron syntactic foam, three-dimensional cubic RVE models with edge lengths L of 25, 35, 45, 55, 65, and 85 μm were generated. The hollow glass microsphere was idealized to consist of a perfect spherical pore and a shell with a constant thickness of 0.806 μm. Three different shell diameters of 8, 12, and 18 μm were introduced in order to take into account the variation in the shell thickness ratio in the models. The volume fraction of the microspheres was approximately 40.8% in all the models. The microspheres were inserted into cubical RVE boxes using the random sequential adsorption (RSA) algorithm [26]. The RSA algorithm involved adding the microspheres sequentially to the cubic volumes by randomly generating the center point of each microsphere. During the algorithm, if newly generated candidate microspheres overlapped with any previously generated ones, they were deleted. A 5% diameter of the microspheres was imposed as a minimum neighbor distance between each microsphere shell to overcome the practical limitations involved in creating adequate meshes in the matrix between the microspheres. A schematic flow chart of the algorithm is shown in Figure 2. In addition, the algorithm was modified to add the microspheres in order of diameter, from the largest to the smallest. For instance, if an initial algorithm for microspheres with a diameter of 18 μm was completed, then a new algorithm for microspheres with a diameter of 12 μm started. All procedures ended when the algorithm for the 8 μm diameter was completed. To avoid the wall effect, which refers to the inability of inclusions to penetrate through the sample boundaries [27], periodic microstructures were assumed. This was where the parts of microspheres intersecting boundary faces of the models were permitted to reappear through the opposite faces of the cubic volume. Three realizations were generated for each of the differently sized RVE models to investigate statistical errors of apparent responses between the models. Figure 3 depicts typical generated RVE models with different edge lengths. The RVE models with L = 25, 35, 45, 55, 65, and 85 μm embedded 6, 12, 25, 50, 82, and 182 microspheres, respectively. 

### 3.2. Model Implementation

**Figure 4:**
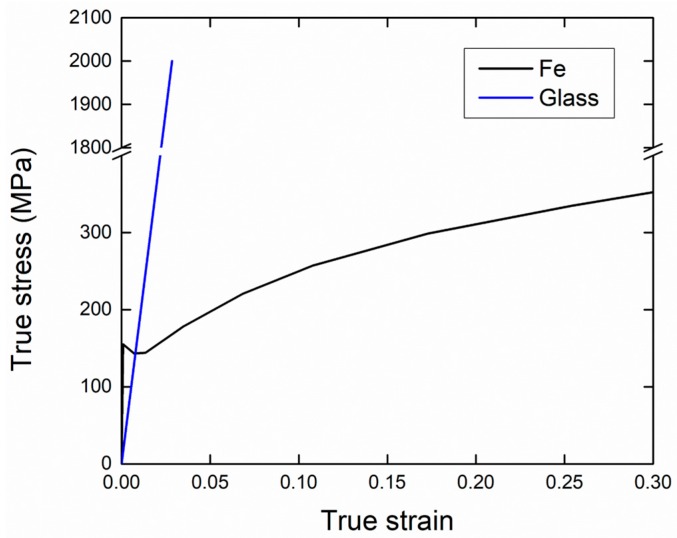
True stress–strain curves of the iron and glass [24,28] used for input data.

The stress–strain curves and properties of the iron and soda-lime borosilicate glass [24,28] used for the analysis are shown in Table 1 and Figure 4. The stress–strain curve of the iron was obtained using the same procedure as the tensile test conducted for the syntactic foam. The deformation behavior of the glass was set to be isotropic and linear elastic. After the tensile strength was applied, the glass fractured in a brittle manner, characterized by Mode I and Mode II failure mechanisms. After maximum principal stress exceeded the tensile strength of the glass, crack initiations were detected using the Rankine criterion (Mode I). Then, stress dropped with the reduction of the shear modulus (Mode II), and the fractures of the glass were consequently visualized via the deletion of element technique [29]. Use of proper tensile strength of the glass is very important because this can influence the overall deformation behavior of the syntactic foam. However, the direct measurement of the strength in experiments is difficult. Since the experimental data on the tensile strength was absent, the strengths from 500 to 2000 MPa were tested, and a proper value was selected by investigating the stress–strain responses. Perfectly bonded matrix/microsphere interfaces were assumed.

Two different boundary conditions (BCs) were used: the static uniform boundary condition (SUBC) and the orthogonal mixed boundary condition (OMBC) [30]. The Hill equation in the conventional volume average form is as follows [31]:

(1) 〈σ:ε〉=〈σ〉 :〈ε〉

where σ and ε are the stress and strain tensors, respectively. The bracket 〈 〉 denotes a volume average of the variable. This equation implies that the average of the product of the stress and strain tensors at micro level equals the product of their averages at a macro level. Using Gauss’ theorem, this condition for a finite body can be written explicitly as [32]

(2) $$\underset{\Gamma }{\int }\left(t\left(x\right)-\langle \sigma \rangle n\right)\cdot \left(u\left(x\right)-\langle \varepsilon \rangle x\right)d\Gamma =0$$

where Γ is the boundary of a volume element V, and t, u, n, and x are the traction, displacement, normal, and position vectors, respectively. The type of the BCs can be classified with respect to the fluctuation term, which is derived from the decomposition of the displacement vector u into a homogeneous deformation $\bar{u}$ and a fluctuation field $\tilde{u}$ ($u=\bar{u}+\tilde{u}$) along the boundaries of V. Based on these equations, one can classify the SUBC and OMBC as

(3) $$t\left(x\right)=\bar{\sigma }n$$

for the SUBC, in which the traction vector t is prescribed at the boundary, and

(4) $$\left(t\left(x\right)-\bar{\sigma }n\right)\cdot \left(u\left(x\right)-\bar{\varepsilon }x\right)=0,\;\tilde{u}=0$$

for the OMBC, in which the loading vector imposed at the boundary contains the components of both the forces and the displacements. In combination with each BC, a uniaxial tensile strain of 7% was imposed on the models. For the SUBC, the tensile strain was applied to one face of each volume element, while the homogeneous deformation $\bar{u}$ on the opposite face was fixed along the loading direction, and all other faces were free from forces. In case of the OMBC, the same procedures were applied, but $\tilde{u}=0$ was set for all the faces.

ABAQUS/Explicit was used to mesh the models, and simulate mechanical responses under macroscopic uniaxial tension. To consider the bending of microsphere shell correctly, several solid elements in the thickness direction should be introduced. However, if the many elements along the thickness with the element deletion technique are used, the elements in contact with the matrix are deleted first. The remaining elements are separated from the matrix and move without constraints, which causes severe contact problems. To suppress these problems in this study, one layer of the high-ordered shell element, SC6R, with five integration points, was used for the microspheres. The single layer of this element can consider the bending effects correctly and cause no contact problems. Then, the matrix was meshed using the four-node tetrahedron element C3D4, which matched the nodes of the existing elements. Mass scaling was applied during the analysis to reduce computation costs, through which acceptable mass scaling value, time increment, and load speed were found by trial and error processes. The energy history was observed to evaluate an appropriate response of the analysis.

## 4. Result and Discussion

### 4.1. Determination of RVE

**Figure 5:**
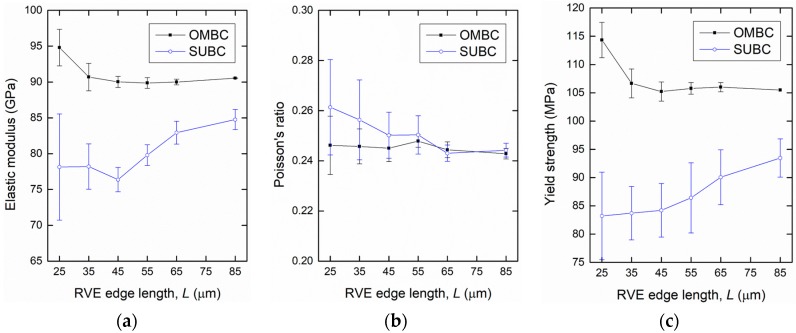
(**a**) Elastic moduli; (**b**) Poisson’s ratios; and (**c**) yield strengths obtained with the representative volume element (RVE) models subjected to different boundary conditions as a function of their edge lengths. Black lines with rectangular marks represent the orthogonal mixed boundary condition (OMBC). Blue lines with circular marks represent the static uniform boundary condition (SUBC).

To determine an appropriate size of the RVE, changes in mechanical responses owing to different numbers of constituents in the model and applied BCs should be considered [27,33,34]. The RVE size can be determined when apparent properties approach specific values with increasing size. In addition, applying different BCs on the RVE boundaries with free faces (in 3D) of particle/matrix interfaces causes force and displacement fluctuations. When these fluctuated regions possess only a small portion compared to the whole model, the model can be representative by neglecting this effect [30,35]. Figure 5 shows the apparent elastic moduli, Poisson’s ratios, and yield strengths with their errors obtained from realizations under the SUBC and OMBC at each RVE edge length. The yield strengths were calculated by inputting 600 MPa for the tensile strength of the glass. At the small model size under both BCs, large discrepancies between the properties were observed. As the size increased, however, these properties converged to each specific value with decreasing deviations. For the L = 55 μm model, the errors of the elastic modulus, Poisson’s ratio, and yield strength were 0.9%, 1.0%, and 1.0%, respectively, for the OMBC. These values are acceptable compared to the previous study, where an error of less than 2% was used as a criterion [36]. It is obvious that the effect of the different microstructures between the realizations on the properties becomes insignificant as the size increases. Moreover, a decrease in microstructure heterogeneity suggested that the isotropy and statistical homogeneity of the models were achieved by choosing a large model for the iron syntactic foam with glass microspheres.

The differences in properties between the BCs also decreased with the increase of the RVE size. The OMBC and SUBC took upper and lower bounds for the properties. However, the elastic modulus and yield strength from the SUBC had a tendency to increase, and the deviations of the properties for the SUBC were larger than those for the OMBC across all sizes. This is because of the characteristic of the SUBC, which allows the lateral boundary faces to move freely without constraints. The decrease in the difference in properties between the BCs implies a decrease of the fluctuated zone effect on the properties. Based on the results from the statistical determination of the RVE size associated with the BCs, a model with an edge length of 55 μm was selected for a proper RVE size. Although the larger-sized models could provide better estimations with respect to the properties, the L = 55 μm model is more efficient when considering the computation cost. Therefore, the L = 55 μm model was selected for the RVE and was used for subsequent simulation and analysis.

When the size of the model is insufficiently large to be an RVE, it is known that the periodic boundary condition (PBC) gives the most similar values to effective properties as compared to the OMBC and SUBC. As previously stated, the OMBC and SUBC take upper and lower bounds, respectively. However, as the size increases, the size of the model to be an RVE becomes different depending on the applied BCs. It is known that the PBC presents the effective property at the smallest size, and OMBC and SUBC show the effective properties in a sequence as the size increases [37]. When the model is large enough, the apparent properties converge to the specific values regardless of the BCs. Hence, if the isotropic properties without deviations irrespective of the realizations are obtained with the OMBC, the OMBC can be used for estimating the effective properties. In addition, in contrast to the PBC and SUBC, the OMBC has an advantage in describing Poisson’s effect directly. Therefore, only the OMBC was used, which presented better estimations than the SUBC.

In order to investigate the accuracy and reliability of the RVE model, the elastic moduli from the RVE, analytical models, and experiments were compared, as shown in Table 2. The modulus of the L = 55 μm model was chosen for the numerical result. The Hashin–Shtrikman (HS_upper_) and Voigt–Reuss (VR_upper_) upper bounds, as well as the composite sphere-based self-consistent scheme (CS-based S-C), were used for the analytical model to calculate the modulus [8,11,28]. As can be seen in Table 2, the RVE model more closely reproduced the experimental findings, whereas the analytical models overestimated the elastic modulus. Since the analytical models cannot directly consider microstructural features such as spatial distributions of the microspheres and microsphere/matrix interactions during deformation, the proposed RVE model is more effective in estimating the elastic modulus than the traditional models. Among the analytical models, the CS-based S-C estimated the most accurate modulus.

**Figure 6:**
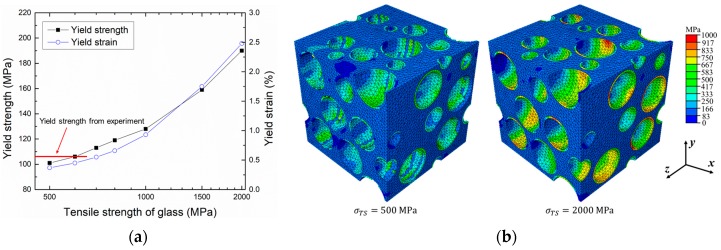
(**a**) Effect of tensile strength of the glass on the yield strength and strain of the iron syntactic foam; (**b**) Equivalent stress fields of model at 0.7% ***x***-axial strain using 500 and 2000 MPa for the tensile strength of the glass. $\sigma_{TS}$ denotes the tensile strength of the glass.

Figure 6a shows changes in yield strength and strain of the L = 55 μm RVE model when tensile strengths of the glass ranging from 500 to 2000 MPa were used. The numerical results suggest that the tensile strength of the glass affects deformation behaviors of the iron syntactic foam. When the strength of the glass was high, the brittle fracture of the glass occurred later, and yield strength and elongation of the syntactic foam increased because the microsphere could bear a great amount of stress. In contrast, the low strength of the glass led to an early brittle fracture of the glass and decreased the yield strength and elongation of the syntactic foam. This effect is clearly indicated in Figure 6b, where deformed and fractured geometries as well as equivalent stress fields of models using the tensile strength of the glass with 500 and 2000 MPa are compared. In the figure, the shape of the microspheres is clearly seen with 2000 MPa, while with 500 MPa, fractured and fragmented shells were observed. It also can be seen that the stress in the microspheres is much higher with 2000 MPa than with 500 MPa, indicating different capacities for load bearing. The elastic properties, however, were not affected by the tensile strength of the glass in the considered range. Compared with experimental results, the yield strength was in agreement with the numerical results when the tensile strength of the glass with 600 MPa was used. It should be noted that this tensile strength of the glass is lower than the strength used by Wei (700 MPa) [38] and Bardella (783 MPa) [39], although they considered the glass microspheres in an epoxy matrix. The low strength can be attributed to the high processing temperature, which may induce changes in the microsphere shape or the glass properties.

**Figure 7:**
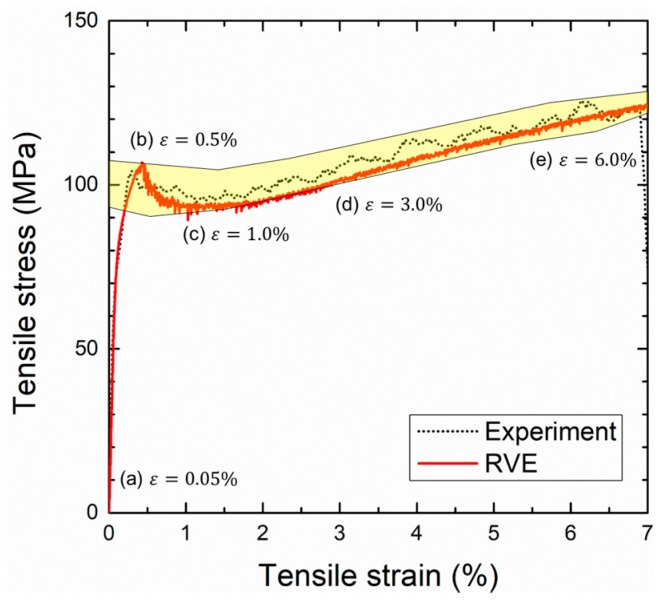
Typical stress–strain curves obtained by the experiment and representative volume element (RVE) model. The yellow-colored region indicates the deviation of the experimental results.

Figure 7 shows the typical stress–strain curves obtained from the L = 55 μm RVE model with the glass tensile strength of 600 MPa and the experiment. The yellow-colored area indicates deviation of the curves from the experiments. The elastic and plastic regimes predicted by the RVE model are in a good agreement with the experimental result, while the yield strength was slightly overestimated and was out of the experimental bounds. These agreements mean that the RVE model, generated by the proposed modeling technique with prescribed material models, accurately describes the microstructure of the syntactic foam.

### 4.2. Tensile Deformation Behaviors

**Figure 8:**
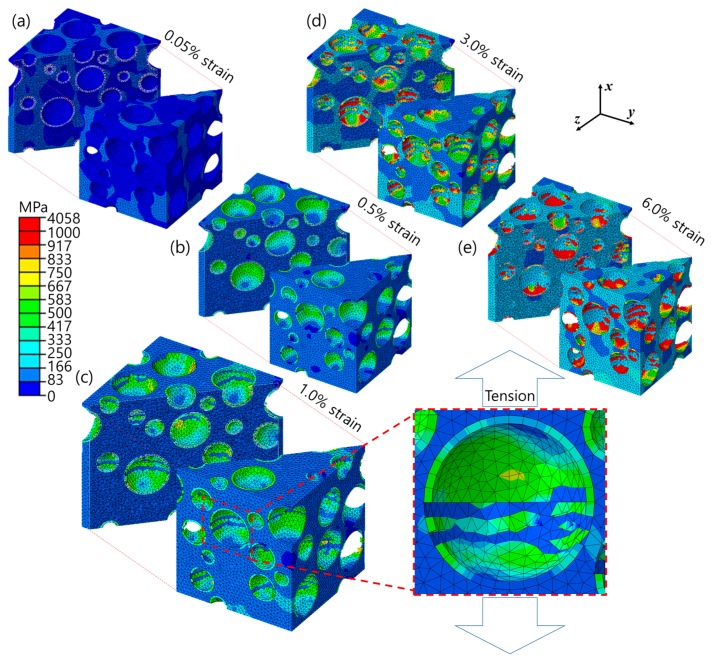
Equivalent stress fields of the L = 55 μm RVE model subjected to ***x***-axial tensile strain of (**a**) 0.05%; (**b**) 0.5%; (**c**) 1.0%; (**d**) 3.0%; and (**e**) 6.0%.

Figure 8 shows the equivalent stress fields of the RVE model under ***x***-axial tensile load at corresponding strains (a–e) indicated in the stress–strain curve of Figure 7. It was interesting that the greater stress first took place at the matrix rather than at the microspheres in the early stage of elastic deformation (Figure 8a), which was unusual for conventional composite materials. The conventional composites mostly consist of matrix and reinforcements with low and high elastic moduli, respectively, so that the reinforcements bear the load and increase the stiffness. In the case where the iron syntactic foams had the iron with a high modulus and the glass with a low modulus, the stiffness-reinforcing effect by the microspheres was small, and the matrix bore most of the load. This is a reason for the stress that developed at the narrow solid areas of the matrix between the microspheres. The applied load was gradually transferred to the microspheres during elastic deformation. To evolve the plasticity in the matrix, more loads should be applied to the iron syntactic foam because of the load-sharing by the microspheres. This is a possible reason why the iron syntactic foams have higher yield strength compared to the conventional iron foams without microspheres. At the yield point (Figure 8b), the brittle fractures of the microspheres started along a direction that was perpendicular to the loading. As seen in Figure 8c, most of the microspheres were fractured and/or fragmented into more than two parts. Therefore, the sudden drop and oscillation of the curve with a 0.5–1.0% strain in Figure 7 were thought to occur due to the continuous fractures of the microspheres. With increasing strain, the microspheres were more fractured, and the magnitude of stress in the matrix increased.

**Figure 9:**
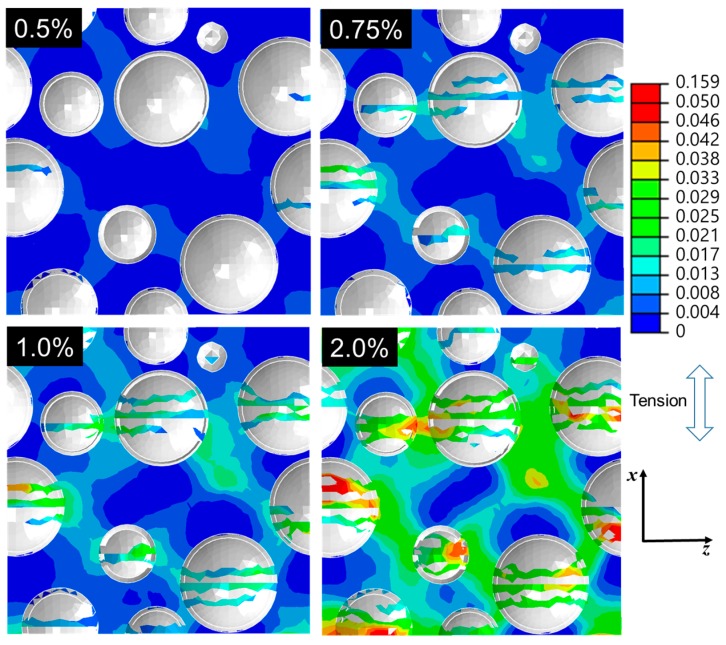
Plastic strain fields of a cross section of the L = 55 μm RVE model subjected to ***x***-axial tensile strain from 0.5% to 2.0%. The microspheres were colored in gray because no plastic deformation occurred.

Figure 9 shows distributions of the plastic strain for the cross section of the same model used in Figure 8. The global plasticity occurred before the fracture of the microspheres. With the continuous fractures of the microspheres, it is clearly seen that the localized plastic deformation took place at the matrix adjacent to the fractured microspheres. 

### 4.3. Discussion

Based on the results presented in Figure 7, Figure 8 and Figure 9, we suggested tensile deformation mechanisms of the iron syntactic foam with glass microspheres. The mechanisms can be divided into three parts. The first is a regime before the yield point. At the very early stage of the (macroscopic) elastic deformation, both the matrix and microspheres suffer the loads elastically until reaching a macroscopic strain of approximately 0.065%. While this matrix carries more loads due to its higher stiffness than the microspheres, after this strain is reached, localized micro-plastic deformations occur within said matrix. The micro-plasticity in the matrix induces load transfer from said matrix to the microspheres. Consequently, near the (macroscopic) yield point, microspheres carry a significant amount of the applied load. At this stage, the global plasticity already takes place in the matrix, although the behavior of the syntactic foam is still apparently elastic. The second part is from the yield point to before a strength-increasing point. At the yield point, the fracture of the microspheres begins. The continuous fractures cause a drop in overall strength. As with the deformation processes, a strain-hardening effect of the matrix compensates the strength decrease due to the fracture of the microspheres, so that the strength remains constant. The last part is the remaining regime. The strength increases continuously because of the larger strain-hardening effect of the matrix.

**Figure 10:**
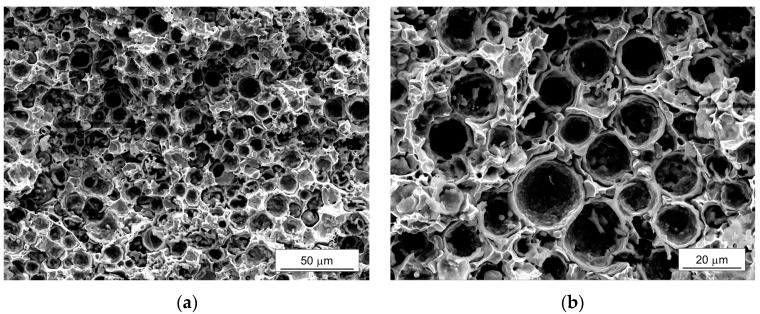
Fracture surfaces of the iron syntactic foam with 5 wt % hollow glass microspheres after the tensile test observed by scanning electron microscope: (**a**) Low magnification; and (**b**) high magnification.

Figure 10 shows the fracture surfaces of the iron syntactic foam with a 5 wt % glass microsphere after the tensile test where three types of the fractures are seen. The ductile fractures (dimples) in the matrix were observed in the proximity of the brittle fractured microsphere shells. The fractured microspheres were mostly bonded to the iron matrix. Partial gaps between the microspheres and matrix, however, indicated that minor interfacial debonding existed. When considering the fracture geometries of the deformed RVE model at 6% strain in Figure 8d, the results from the RVE are somewhat unrealistic compared to these experimental findings. Up to a 2% strain, as shown in Figure 9, the shapes of the fractured microspheres are similar to the experimental result. After 3%, as shown in Figure 8c–d, more microspheres were fractured and numerically deleted, and thus fragmented. These fragmented parts continuously suffered the load with increasing strain. However, in the actual fracture surfaces, the hemispherical shapes of the fractured microspheres were preserved with their shells. This means that, at high strain, the iron syntactic foam will be deformed by the other mechanisms.

It is known that the possible deformation mechanisms of particle-reinforced metal matrix composites can be categorized into three, depending on states between the particle, matrix, and interface [40]. When the interface is weak, the initiation of the crack starts there. In the case where the particle strength is low, the particle will be loaded to the crack. If both the interface and the particle are strong, a void will form in the matrix and develop to the crack. Additionally, it has been reported that a clustering of the microspheres can induce debonding in the case of the syntactic foam [41]. The proposed RVE model in this study considered the deformation of the matrix and microspheres, and similar results to the actual fracture surface, until a medium strain was reached, have been found. One possible type of fracture that was not been taken into account is that caused by interfacial debonding. To retain the spherical shapes of the microspheres, the debonding between the matrix and microspheres during deformation seems necessary so that the load transfer from the matrix to the microspheres can be avoided. Previous studies have reported that the debonding of the microspheres during the tensile deformation of the metal syntactic foams occurred in the combination of microsphere fracture and crack propagation through the matrix [4,17,41,42]. Therefore, the remaining discussion will briefly focus on the interfacial debonding behavior, which can improve the numerical results of the proposed RVE model.

**Figure 11:**
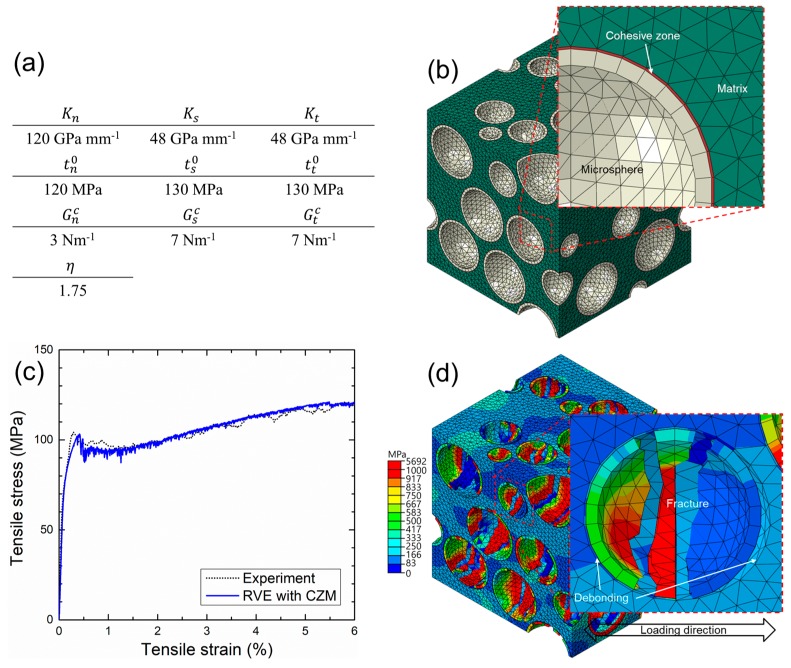
(**a**) Input parameters for cohesive zone model; (**b**) Geometry of representative volume element (RVE) with cohesive elements; (**c**) Stress–strain curve of numerical results compared with experiments; (**d**) Equivalent stress field near a debonded microsphere at 6% strain.

Numerical studies on the debonding of particles or microspheres in composites have typically used three different techniques: control of the contact condition between the matrix and particles, introduction of a cap crack on the interface, and use of a cohesive zone model (CZM) by assuming the existence of an intermediate phase between the matrix and particles [16,18,19,20,43]. In this study, to improve the proposed RVE model, the feasibility of the CZM was tested to realize the interfacial debonding of the iron syntactic foam with hollow glass microspheres. Explanations on cohesive elements and models are summarized in [16]. Bilinear traction–separation law was used for a governing equation with a stress-determined damage initiation criterion and an energy-based damage evolution criterion proposed by Benzeggagh and Kenane [44]. Although the parameters used for the equations should be obtained by numerous experiments, the experimental result and data from the literature were absent. Therefore, some parameters used by Yu [16] and arbitrary values obtained by trial and error processes were used, since the purpose of this test was to examine a feasibility of the interfacial debonding in iron syntactic foams. The used parameters and model, and the numerical results, are shown in Figure 11. The strain–stress curve from the RVE associated with CZM agreed with our experimental results. A decrease in the strain hardening rate at 5.5% strain was caused by the debonding of the microspheres. Figure 11d indicates the partial debonding of the fractured microspheres. Similar to the fracture surfaces in Figure 10, most of the microspheres retained their shapes without fragmentation. From the results, it was possible to numerically determine the microsphere debonding.

The RVE model proposed here considers the fracture effect of the microspheres, which is helpful to better understand the elastoplastic deformation behavior of the iron syntactic foam. The model can be easily adopted for syntactic foams consisting of the matrix with high stiffness and low strength, and the microsphere with low stiffness and high strength, where an early fracture of the microsphere is expected. Moreover, the effects of different microstructural parameters such as the size and shell thickness of the microsphere can be investigated by the model. It was also found that the realization of the interfacial debonding between the matrix and microsphere improved the model to describe the actual fracture behavior of the iron syntactic foam. However, since the parameters used for interfacial debonding were arbitrarily selected, accurate values should be found using proper experiments. In addition, it is worth performing more research to improve the RVE model by considering the failure of the matrix in the future.

Additionally, it should be noted that the element deletion technique used in this study has a strong dependency on mesh density. The mechanical responses and deformed shape of the model can vary with the mesh density. As the size of the element decreases, this dependency becomes weak. Therefore, careful use of this technique, with an investigation of the change in mechanical responses in terms of the mesh density to determine the proper element size, will be needed. If other techniques, such as the introduction of a structural criterion for the failure of the glass [21], are undertaken, there will be no need to perform dynamic explicit analysis.

## 5. Summary and Outlooks

In this study, a three-dimensional finite element model was developed to represent the tensile deformation behaviors of the iron syntactic foam with 5 wt % hollow glass microspheres using the RVE approach and the RSA algorithm. By statistically evaluating mechanical responses in terms of the RVE size and applied BCs, a proper RVE with L = 55 μm was selected. The selected RVE showed the results having a better agreement with the experimental findings than the analytical models. Tensile deformation behaviors of the iron syntactic foam were investigated using the RVE model. The matrix bore more loads than the microspheres at the beginning of the elastic deformation, and the loads were transferred gradually to the microspheres. The yielding of the iron syntactic foam occurred at the beginning of the microsphere fracture. The fractures of the microspheres caused a drop in overall strength. The plastic deformation evolved in the matrix adjacent to the fractured regions. When strength was increased through a strain-hardening effect, most of the microspheres fractured into more than two parts. To improve the accuracy of the proposed model, the feasibility of the interfacial debonding was tested. Consideration of the debonding showed more practical results compared to the experimental findings. The proposed RVE model and its modeling techniques can be used to investigate the elastoplastic responses of not only syntactic foams but also composites with matrices with high stiffness and low strength, as well as reinforcements with low stiffness and high strength, whose early fracture is expected. The proposed RVE model can also be used for the design of new materials.

## Figures and Tables

**Table 1:** Material properties used for numerical analysis.

Material	ρ (kg·m^−3^)	*E* (GPa)	υ	σ (MPa)
Iron	7870	198.0	0.28	155
Glass [24,28]	2500	70.1	0.23	500–2000

**Table 2:** Comparison of elastic moduli with analytical models and experimental results. HS_upper_, VR_upper_, and CS-based S-C denote the Hashin–Shtrikman and Voight–Reuss upper bounds, and the composite sphere-based self-consistent scheme, respectively [8,11,28].

Results	RVE	Experimental	HS_upper_	VR_upper_	CS-Based S-C
*E* (GPa)	89.9	88.9	91.2	124.5	90.5
